# The Korean Social Life, Health and Aging Project-Health Examination Cohort

**DOI:** 10.4178/epih/e2014003

**Published:** 2014-05-13

**Authors:** Ju-Mi Lee, Won Joon Lee, Hyeon Chang Kim, Wungrak Choi, Jina Lee, Kiho Sung, Sang Hui Chu, Yeong-Ran Park, Yoosik Youm

**Affiliations:** 1 Department of Preventive Medicine, Yonsei University College of Medicine, Seoul, Korea; 2 Cardiovascular and Metabolic Diseases Etiology Research Center, Yonsei University College of Medicine, Seoul, Korea; 3 Institute of Vision Research, Department of Ophthalmology, Yonsei University College of Medicine, Seoul, Korea; 4 Department of Sociology, Yonsei University College of Social Sciences, Seoul, Korea; 5 Department of Clinical Nursing Science, Yonsei University College of Nursing, Seoul, Korea; 6 Division of Silver Industry, Kangnam University, Yongin, Korea

**Keywords:** Cohort studies, Social determinants of health, Aged, Rural population, Korea

## Abstract

The Korean Social Life, Health, and Aging Project (KSHAP) is a population-based longitudinal study of health determinants among elderly Koreans. The target population of the KSHAP are people aged 60 years or older and their spouses living in a rural community of Korea. A complete enumeration survey was conducted in the first wave of the KSHAP on 94.7% (814 of 860) of the target population between December 2011 and July 2012. The KSHAP-Health Examination (KSHAP-HE) cohort consists of 698 people who completed additional health examinations at a public health center (n=533) or at their home (n=165). Face-to-face questionnaires were used to interview participants on their demographics, social network characteristics, medical history, health behaviors, cognitive function, and depression symptoms. Health center examinations included anthropometric measures, body impedance analysis, resting blood pressure measurement, radial artery tonometry, bone densitometry, the timed up-and-go test, and fasting blood analysis. However, only anthropometric measures, blood pressure measurement, and non-fasting blood analysis were available for home health examinations. Collaboration is encouraged and access to the KSHAP baseline data will be available via the website of the Korean Social Science Data Archive (http://www.kossda.or.kr).

## INTRODUCTION

The Korean Social Life, Health, and Aging Project (KSHAP) began by benchmarking from the US National Social Life, Health, and Aging Project (NSHAP). The NSHAP interviewed 3,005 community-dwelling adults aged 57 to 85 years across the US [1]. The NSHAP collected ego-centric social network data with a module that allows each respondent to identify the socio-demographic information of their network members and the relationships among them [2]. Using the NSHAP data, researchers have found that some types of social networks were associated with various health-related dimensions such as subjective well-being [3], depressive symptoms [4], hypertension [5], health-related behaviors [6], health-care utilization [7], and others.

The KSHAP is a longitudinal population-based study aiming to understand the current status, trends, and determinants of health among older, community-dwelling Koreans. Detailed information on the KSHAP study design will be published elsewhere. Briefly, the purpose of the KSHAP is to examine physical health, emotional health, cognitive function, health behaviors, medical service use, social connectedness, and relationship quality as well as assess any interactions within these variables and collect follow-up data. The KSHAP-Health Examination (KSHAP-HE) cohort additionally aims to measure anthropometric variables, blood biomarkers, blood pressure, mobility function, and bone density to assess determinants of cardiovasCorrespondencecular and metabolic health among elderly Koreans. The KSHAP-HE study was designed using a multi-disciplinary approach including a social network analysis, questionnaire interview, physical examination, functional assessment, and biomarker analysis to comprehensively understand social, emotional, and physical health.

## STUDY PARTICIPANTS

The KSHAP aimed to recruit the entire population (not a sample) of adults aged 60 years or older and their spouses living in within one township in Gangwha Island, Korea. This township is a typical, rural, Korean village where farming is the main industry. As of January 2013, the total population was estimated as 1,864 people and 871 families. With the aid of township officers and after performing a pilot study, a total of 860 people were identified as the target population of KSHAP. Of these 860 adults, face-to-face interviews were performed on 814 people from December 2011 to March 2012 (94% response rate). All KSHAP participants were also invited to participate in a health examination at the local public health center, and 533 adults (65.5%) completed the examination. An additional 165 people (20.3%) participated in home health examinations from March through July 2012. In total, data on 698 people (85.7% of KSHAP participants; 81.1% of target population) who completed health examinations were collected for the KSHAP-HE cohort.

## ETHICAL CONSIDERATIONS

The institutional review board (IRB) of Yonsei University approved this study (YUIRB-2011-012-01). All participants were informed of their right to withdraw from the study at any point with no penalty, and informed consent was provided. All scientific data were formulated as anonymous and encrypted. The anonymous baseline dataset was registered with the Korean Social Science Data Archive.

## MEASUREMENTS

The KSHAP-HE cohort plans to collect follow-up health examination data between 2015 and 2017. Follow-up examinations will repeat the baseline measurements to observe any 5-year changes to physical health, emotional health, cognitive function, health behaviors, and social network characteristics. In addition to the active follow-up plan, information on newly-diagnosed diseases and deaths will be collected from self-reported and family-reported data as well as through the National Health Insurance claims database and National Mortality Database.

## QUESTIONNAIRE INTERVIEWS

The interviews were conducted at the public health center or respondents’ homes for an average of 48 minutes. Participants were interviewed by trained personnel using standardized questionnaires according to the pre-determined protocol. In addition, the entire interview process was continuously monitored by the designated field director.

Participants were asked about their socio-demographic factors, medical history, and health behaviors. Socio-demographic measures included education, occupation, marriage, household income, religion, the social relationship within their family and community, and social support received from others. Medical history included hypertension and hypertension management, diabetes and diabetes management, dyslipidemia and dyslipidemia management, metabolic syndrome, osteoporosis, fractures, incidents of falls, cancer, stroke, heart diseases, arthritis, lung diseases, eye disease, hepatitis, depression, urinary incontinence, and prostate enlargement. Health behaviors included cigarette smoking, alcohol drinking, sleep duration, and frequency of receiving vaccination and health screenings. Depression symptoms were assessed by the Center for Epidemiologic Studies-Depression Scale [8]. In addition, questions about stress level, overall life satisfaction, thoughts of suicide, and suicide attempts were asked. Cognitive function was assessed using the Korean version of Mini Mental State Examination for Dementia Screening [9-11]. Overall health status was assessed using the 12-item Short Form Health Survey, which was validated in a previous study [12] and translated for use in Korea [13,14].

Social network characteristics were assessed using the Korean version of the social network survey, which is an identical questionnaire to the NSHAP from the US. Prior to the social network survey, we constructed a comprehensive list of the target population with the aid of the community office and a pilot study. With the complete social network, we were able to perform a global network analysis. A detailed description of the methods and results of the social network analysis will be published elsewhere. Typically, psychological measures are the main focus of validation when translating questionnaires; however, questionnaires that collect data on social network measures have been applied across various societies since they are supposed to measure actual behaviors not attitudes or beliefs. Since Burt proposed a name generator to measure social networks to be used in the General Social Survey in the US [15], a similar but less elaborate version of this kind of social network questionnaire has been used in the Korean General Social Survey since 2003.

## HEALTH EXAMINATIONS

At the public health center, collected measurements included body composition, resting blood pressure, radial pulse wave analysis, bone densitometry, the timed up-and-go (TUG) test, and fasting blood analysis. Standing height was measured to the nearest 0.1 cm using a stadiometer and body weight was measured to the nearest 0.1 kg on a digital scale. Body mass index (BMI) was calculated as body weight in kilograms divided by height in meters squared. Body composition data collected body fat mass, percent body fat, lean mass, fat free mass, skeletal muscle mass, and appendicular fat free mass, which were all estimated using the bioelectric impedance analyzer (InBody 370; Biospace Co. Ltd., Seoul, Korea). Resting systolic and diastolic blood pressure, mean arterial pressure, and pulse rate were measured twice with the oscilloscopic method using an automatic sphygmomanometer (CareScape V100; GE Healthcare Medical Systems Information Technologies Inc., Milwaukee, WI, USA). Prior to each measurement, all participants rested for at least five minutes in a seated position, and the cuff was adapted to the circumference of their right upper arm. If the first and second measurements differed by ≥10 mmHg, either for systolic or diastolic pressure, the additional blood pressure measurements were performed, and the average of the last two measurements was used to determine the blood pressure level. In addition, the radial augmentation index and central blood pressure measurements were estimated using an automated radial pulse waveform analyzer (HEM-9000AI; Omron Healthcare, Kyoto, Japan). Mobility function was assessed by a TUG test. This test measures the amount of time, to the nearest second, taken to rise from a chair, walk 3 meters to the end of a pre-arranged line, and then return to the chair and sit down. Blood samples were collected from the antecubital vein after at least an 8 hour fast. Collected blood samples were analyzed at a central research laboratory for complete blood count, glucose, insulin, total cholesterol, high-density lipoprotein cholesterol, triglycerides, protein, albumin, aspartate aminotransferase, alanine aminotransferase, urea nitrogen, creatinine, and C-reactive protein. Baseline assessments performed in the KSHAP-HE cohort are summarized in Table 1.

## BASELINE CHARACTERISTICS OF THE COHORT

Table 2 summarizes the baseline characteristics acquired from questionnaire interviews. The mean age was 72.4 years for the total population (n=698), 72.9 years among males (n=286), and 72.1 years among females (n=412). Male participants had higher levels of education and were more likely to smoke cigarettes and drink alcohol than were female participants. However, diagnoses of hypertension and osteoporosis were more frequent among females than males. The results of the health examinations at each site are shown in Tables 3 and 4.

As of March 2014, two articles have been published in peer-reviewed journals analyzing baseline KSHAP-HE cohort data. A cross-sectional analysis of 657 KSHAP-HE participants (aged 60 years or older) indicated that social network characteristics differed according to BMI among males (n=273) and females (n=384). After adjusting for potential confounders, a larger but coarse social network size was significantly associated with a higher BMI among men (p=0.037), while a lower communication frequency was associated with a higher BMI among women (p=0.049). However, network density and size were not associated with BMI among women, and communication frequency was not associated with BMI among men [16]. These findings suggest that social network structure (network size and density) and activation (communication or meeting frequency) may affect physical health among men and women differently. We also are planning to analyze gender differences in the association between social network characteristics and emotional health in a future study.

Another cross-sectional study analyzed only participants who had completed health examinations at the public health center and observed an independent association between decreased muscle mass and increased arterial wall stiffness [17]. Appendicular skeletal muscle mass was inversely associated with the augmentation index, an indicator of arterial wall stiffness, even after adjustment for age, BMI, systolic blood pressure, total cholesterol, high-density lipoprotein-cholesterol, fasting glucose, insulin, smoking, and alcohol intake [17].

## STRENGTHS AND WEAKNESSES

The most important strength of this cohort is the multidisciplinary assessment of health and its determinants. The baseline examinations include not only a general health questionnaire and physical examination but also an analysis of social network, blood biomarkers, and some functional tests. A second strength is that this cohort is based within one community and highly representative of this community. Collaboration with the local township offices, public health center, and senior societies as well as the ability to visit homes within the community enabled the high response rate of 94.7% for the KSHAP study and 81.1% for the KSHAP-HE cohort study. Third, we attempted to enroll all respondents’ spouses. In total, 235 couples completed both the baseline survey and physical examination. This allows for a future analysis of couple-matched data within this population. Fourth, the KSHAP dataset collected social network data using many identical questions used in the NSHAP in the US for the purpose of international comparison study.

The first weakness of this cohort is a lack of external validation. Although this is a community-based cohort with a high response rate, the study population is only representative of a single community and not of the general Korean population; however, this community is a typical, rural, Korean village. Further studies are required to investigate older Korean adults living outside of rural areas. Second, our sample size is relatively small (n=698). We are trying to expand recruitment to neighboring communities to increase the statistical power. Third, a limited numbers of physical examinations and laboratory tests were collected at the public health center or at participants’ homes, and no bio-specimens other than serum were stored for future research.

## DATA ACCESSIBILITY

Collaborative research is highly encouraged. The KSHAP anonymous dataset is accessible on the Korean Social Science Data Archive’s website (http://www.kossda.or.kr). Any interested researchers may have access to the data for academic and teaching purposes without applying for permission. However, for publication purposes, collaboration with a KSHAP investigator is strongly recommended to prevent duplicate publications and inconsistent findings. Researchers interested in collaborative work or requiring further information are invited to contact the KSHAP principal investigator, Yoosik Youm, at yoosik@yonsei.ac.kr or the co-principal investigator for the KSHAP-HE cohort, Hyeon Chang Kim, at hckim@yuhs.ac.

## Figures and Tables

**Table 1.** Summary of the baseline data collection of the Korean Social Life, Health and Aging Project-Health Examination Cohort

Categories	Measurements
Socio-demographic	Household membership
data	Household income
	Education
	Current and past occupation
	Social position in the community
	Marital status
	Social support from spouse
	Social support from family members and relatives
	Social support from friends and neighbors
	Intergenerational support
	Social activity involvement
	Welfare service usage
Social network	Complete network analysis
analysis	Ego-centric network analysis
Health behaviors	Cigarette smoking
	Alcohol consumption
	Sleep duration
	Health screening
	Influenza vaccination
Physical health	Self-rated general health (Korean version of SF-12)
	Physician diagnosed diseases
	Falls
Mental health and	Self-rated emotional stress
cognitive function	Suicidal thoughts and/or attempts
	Center for Epidemiologic Studies-Depression Scale
	Life satisfaction
	Mini Mental State Examination for Dementia Screening
Physical examina-	Standing height and body weight
tion and perfor-	Waist, hip, and thigh circumference
mance	Resting brachial blood pressure
	Bio-impedence analysis for body composition study^1^
	Radial pulse wave analysis for augmentation index and central blood pressure^1^
	Ultrasound calcaneus bone densitometry^1^
	Timed-up-and-go test^1^
Blood assays	Serum storage
	Blood cell counts, hemoglobin, hematocrit, MCV, MCH, and MCHC
	Serum protein, albumin, and total bilirubin
	Aspartate and alanine aminotransferase levels
	Blood urea nitrogen and creatinine
	Glucose, insulin
	Total cholesterol, high-density lipoprotein-cholesterol, and triglycerides
	C-reactive protein

MCV, mean corpuscular volume; MCH, mean corpuscular hemoglobin; MCHC, mean corpuscular hemoglobin concentration.

1 Not available for home health examinations.

**Table 2.** Socio-demographics, health behaviors, and known diseases of the cohort members

Variables	Total (n=698)	Men (n=286)	Women (n=412)	p for gender comparison
Age (yr)				
Continuous	72.4 ± 7.9	72.9 ± 7.1	72.1 ± 8.4	0.174
≤59	32 (4.6)	3 (1.1)	29 (7.0)	0.002
60-69	206 (29.5)	85 (29.7)	121 (29.4)	
70-79	346 (49.6)	155 (54.2)	191 (46.4)	
80-89	97 (13.9)	39 (13.6)	58 (14.1)	
≥90	17 (2.4)	4 (1.4)	13 (3.2)	
Education (yr)				
Never	214 (30.7)	53 (18.5)	161 (39.1)	<0.001
≤6	199 (28.5)	100 (35.0)	99 (24.0)	
7-9	64 (9.2)	29 (10.1)	35 (8.5)	
10-12	168 (24.1)	78 (27.3)	90 (21.8)	
≥13	53 (7.6)	26 (9.1)	27 (6.6)	
Marriage				
Currently married	520 (74.5)	262 (91.6)	258 (62.6)	<0.001
Divorced, widowed	173 (24.8)	20 (7.0)	153 (37.1)	
Unmarried	5 (0.7)	4 (1.4)	1 (0.2)	
Employment				
Employed	412 (59.0)	203 (71.0)	209 (50.7)	<0.001
Self-employed	9 (1.3)	4 (1.4)	5 (1.2)	
Unemployed	277 (39.7)	79 (27.6)	198 (48.1)	
Social network characteristics^1^				
In-degree centrality	1.98 ± 1.60	2.15 ± 1.65	1.86 ± 1.55	0.019
Out-degree centrality	2.25 ± 1.32	2.47 ± 1.39	2.10 ± 1.24	<0.001
Communication density	0.98 ± 0.11	0.99 ± 0.06	0.97 ± 0.13	0.049
Friendship density	0.88 ± 0.27	0.89 ± 0.23	0.87 ± 0.29	0.215
Cigarette smoking				
Non-smoker	494 (70.8)	90 (31.5)	404 (98.1)	<0.001
Ex-smoker	120 (17.2	120 (42.0	0 (0.0)	
Current smoker	84 (12.0)	76 (26.6)	8 (1.9)	
Alcoholic consumption				
Non-drinker	465 (66.6)	122 (42.7)	343 (83.3)	<0.001
Drinker (<1/wk)	86 (12.3)	43 (15.0)	43 (10.4)	
Drinker (≥1/wk)	147 (21.1)	121 (42.3)	26 (6.3)	
Physician-diagnosed diseases				
Hypertension	359 (51.4)	130 (45.5)	229 (55.6)	0.009
Diabetes mellitus	130 (18.6)	60 (21.0)	70 (17.0)	0.183
Dyslipidemia	68 (9.8	25 (8.7	43 (10.5	0.445
Osteoporosis/osteopenia	164 (23.5)	20 (7.0)	144 (35.0)	<0.001
Cancer	30 (4.3)	17 (5.9)	13 (3.2)	0.074

Data are presented as mean±standard deviation or mumber (%).

1 Social network characteristics were available for 643 people (270 males and 373 females). In-degree centrality indicates the number of people who chose the respondent as their social partner; Out-degree centrality indicates the number of people the respondent indicated as social partners; Communication density indicates the proportion of all possible pairs of social ties those who reported ever talking to each other; Friendship density indicates the proportion of all possible pairs of social ties those who reported feeling close to each one another.

**Table 3.** Site-specific results of the physical examinations

Variables	Public health center examinations			Home examinations		
	Total (n=533)	Men (n=212)	Women (n=321)	Total (n=165)	Men (n=74)	Women (n=91)
Age (yr)	72.5 ± 7.5	73.0 ± 6.7	72.1 ± 8.0	72.2 ± 9.1	72.6 ± 8.2	71.9 ± 9.8
Weight (kg)	58.1 ± 10.5 (n=531)	62.6 ± 10.3	55.1 ± 9.5 (n=319)	57.3 ± 10.7 (n=155)	61.9 ± 10.3 (n=71)	53.5 ± 9.6 (n=84)
Height (cm)	155.2 ± 9.0 (n=531)	163.1 ± 6.0	150.0 ± 6.4 (n=319)	154.4 ± 10.1 (n=155)	161.8 ± 6.7 (n=70)	148.3 ± 8.2 (n=84)
Body mass index (kg/m²)	24.0 ± 3.4 (n=531)	23.5 ± 3.3	24.3 ± 3.4 (n=319)	23.9 ± 3.4 (n=150)	23.7 ± 3.2 (n=68)	24.0 ± 3.6 (n=82)
Percent body fat (%)	33.5 ± 8.4 (n=518)	28.7 ± 8.3 (n=209)	36.8 ± 6.8 (n=309)			
Systolic BP (mmHg)	132.9 ± 19.6	131.6 ± 19.1	133.7 ± 20.0	138.3 ± 21.2	136.0 ± 21.1	140.1 ± 21.3
Diastolic BP, mmHg	71.4 ± 9.9	73.0 ± 10.2	70.3 ± 9.6	74.2 ± 10.4	73.1 ± 10.4	75.1 ± 10.4
Pulse rate (/min)	69.1 ± 11.25	68.6 ± 11.8	69.4 ± 10.9			
Augmentation index (%)	91.5 ± 12.2 (n=522)	86.9 ± 12.8 (n=208)	94.6 ± 10.8 (n=314)			
T-score at calcaneus bone	-2.07 ± 1.47 (n=525)	-1.57 ± 1.28 (n=211)	-2.41 ± 1.50 (n=313)			
Timed up-and-go test						
<14 s	342 (64.7)	148 (69.8)	194 (61.2)			
≥14 s	187 (35.4)	64 (30.2)	123 (38.8)			

Data are presented as mean±standard deviation or number (%).

BP, blood pressure.

**Table 4.** Site-specific blood assay results

Variables	Public health center examinations			Home examinations		
	Total(n=524)	Men(n=209)	Women(n=315)	Total(n=165)	Men(n=72)	Women(n=91)
Hemoglobin ( g/dL)	13.6 ± 1.3 (n=522)	14.4 ± 1.2	13.1 ± 1.0 (n=313)	12.7 ± 1.5	13.5 ± 1.3	12.0 ± 1.3
Hematocrit (%)	42.4 ± 3.9 (n=522)	44.7 ± 3.7	40.8 ± 3.2 (n=313)	40.0 ± 4.6	42.5 ± 4.0	38.0 ± 4.1
Protein (g/dL)	7.39 ± 0.43	7.41 ± 0.43	7.37 ± 0.43	7.10 ± 0.49	7.04 ± 0.48	7.15 ± 0.49
Albumin (g/dL)	4.41 ± 0.23	4.41 ± 0.24	4.04 ± 0.23	4.14 ± 0.34	4.10 ± 0.32	4.18 ± 0.35
Total bilirubin (g/dL)	0.64 (0.53, 0.80)	0.70 (0.57, 0.92)	0.60 (0.51, 0.73)	0.49 (0.38, 0.70)	0.55 (0.43, 0.78)	0.45 (0.36, 0.61)
Aspartate amiotansferase (U/L)	26 (23, 30)	27 (23, 32)	25 (22, 29)	26 (22, 31)	27 (24, 32)	25 (21, 29)
Alanine aminotransferase (U/L)	20 (16, 27)	22 (17, 29)	19 (16, 26)	19 (14, 24)	20 (17, 26)	16 (13, 21)
Blood urea nitrogen (mg/dL)	15.3 (12.8, 18.1)	16.0 (13.5, 18.7)	14.8 (12.4, 17.6)	17.9 (14.7, 22.5)	19.3 (15.0, 22.5)	17.9 (14.4, 22.8)
Creatinine (mg/dL)	0.93 (0.84, 1.06)	1.05 (0.96, 1.14)	0.86 (0.81, 0.95)	1.03 (0.90, 1.19)	1.14 (0.99, 1.26)	0.93 (0.87, 1.11)
Glucose (mg/dL)	89 (83, 97)	91 (84, 101)	88 (83, 94)	99 (79, 125)	97 (78, 127)	103 (79, 125)
Insulin (uIU/mL)	6.9 (5.6, 8.9)	6.5 (5.1, 8.4)	7.3 (6.0, 9.3)	15.4 (8.2, 28.7)	15.2 (8.6, 25.1)	15.5 (7.8, 31.7)
Total cholesterol (mg/dL)	183 (160, 209)	173 (151, 194)	192 (16, 214)	165 (144, 197)	161 (140, 185)	176 (148, 203)
HDL cholesterol (mg/dL)	50 (44, 60)	50 (43, 57)	51 (44, 61)	45 (38, 54)	44 (37, 52)	46 (38, 54)
Triglycerides (mg/dL)	139 (104, 87)	135 (99, 185)	141 (105, 189)	132 (97, 190)	114 (92, 177)	134 (102, 197)
C-reactive protein (mg/L)	0.87 (0.50, 1.79)	1.18 (0.64, 2.55)	0.79 (0.45, 1.45)	0.72 (0.36, 1.57)	0.78 (0.37, 2.50)	0.68 (0.35, 1.25)

Data are presented as mean±standard deviation or median (25th-75th percentiles).

HDL, high-density lipoprotein.
